# Standardized tensile testing of soft tissue using a 3D printed clamping system

**DOI:** 10.1016/j.ohx.2020.e00159

**Published:** 2020-11-21

**Authors:** Mario Scholze, Sarah Safavi, Kai Chun Li, Benjamin Ondruschka, Michael Werner, Johann Zwirner, Niels Hammer

**Affiliations:** a Institute of Materials Science and Engineering, Chemnitz University of Technology, Chemnitz, Germany; b Department of Clinical and Macroscopic Anatomy, Medical University of Graz, Graz, Austria; c Department of Anatomy, University of Otago, Dunedin, New Zealand; d Department of Oral Rehabilitation, Sir John Walsh Research Institute, Faculty of Dentistry, University of Otago, Dunedin, New Zealand; e Institute of Legal Medicine, University Medical Center Hamburg-Eppendorf, Hamburg, Germany; f Fraunhofer Institute for Machine Tools and Forming Technology IWU, Dresden, Germany; g Institute of Anatomy, University of Leipzig, Leipzig, Germany; h Department of Orthopedic and Trauma Surgery, University of Leipzig, Leipzig, Germany

**Keywords:** 3D printing, Biomechanical testing, Clamps, Soft tissues, Tensile testing

## Abstract

Biomechanical testing of soft tissues forms the backbone in the experimental validation of tissue engineering and for modelling purposes. The standardized testing of soft tissues requires different experimental protocols and fixtures compared to hard tissues or non-biological materials due to their characteristics. Some of the most commonly-used clamping methods for soft tissue testing affect the tissues’ mechanical properties as chemicals are involved to decelerate degradation and autolysis. Moreover, they are unsuitable for standardized and high-throughput testing. Material slippage is also a recurrent unwanted influence on the testing routine with impact on measurement validity. Addressing these issues, this protocol presents a clamping system for simplified testing of biological soft tissues with all necessary components manufactured utilizing 3D printing technology. Templates allow trimming the samples into standardized shapes and sizes while preparation tables facilitate clamping in a fixed distance. The key parts of the system are clamps with a pyramid design, which allow the mounting of biological soft tissues before transferring it into the testing device and minimize material slippage during tensile testing. Flexible holder arms are used to transfer samples from preparation tables into the testing device and simplify positioning. Mechanical testing itself is performed with digital image correlation for precise strain measurements.

## Graphical abstract

**Figure:**
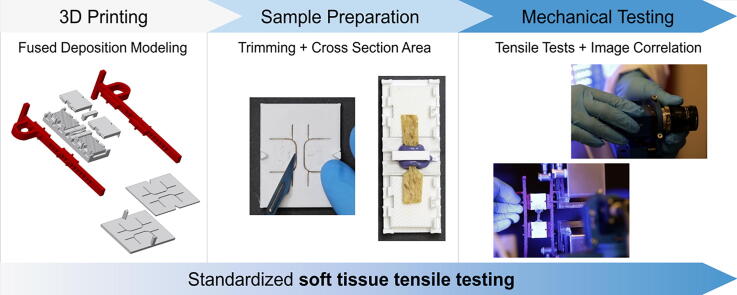

## 1 Hardware in context

Uniaxial tensile testing is a fundamental engineering test and the most commonly used method for obtaining mechanical characteristics of different materials. To standardize this testing procedure, a high number of mainly material-dependent standards has been introduced in the past decades, e.g. for metals [1], ceramics [2], plastics [3], composites [4] or rubber [5]. These standards provide recommendations for experimental setup including sample shapes, testing velocities, data acquisition, and for the subsequent evaluation of the obtained data. Nevertheless, no standard exists for the mechanical testing of biological tissues in general or in detail for human samples so far. As the characterization of human tissue becomes increasingly important for assessing existent or developing new diagnostic, reconstructive and treatment strategies, the application of destructive material testing is inevitable to map human soft tissues properly. Also, developing novel implants and engineered scaffolds to replace injured tissue requires in-depth knowledge of the mechanical properties of the tissue [6] – both in an intact and an injured state. The mechanical characteristics of biological soft tissues including anisotropy, heterogeneity, viscoelasticity, temperature dependence and inter-individual variation make any experimental design challenging [7], [8], [9]. Furthermore, material slippage describing the unintended slipping of samples out of the clamping jaws when loaded is a widely observed issue during testing [10], [11], [12], [13], [14], [15]. Consequently, deviations in strain and force measurements may occur and lead to additional scatter in mechanical properties apart from the expected inter-individual difference of human tissues showing broad variation. To obtain reproducible and consistent results and consequently meaningful information, a sophisticated level of standardization is required for soft tissue material testing. First, a fast and accurate insertion and alignment of the soft tissues in the testing device is required to prevent the tissue from degradation, dehydration or damage of the samples during the setup. Furthermore, a suitable clamping method is necessary to reduce material slippage when testing mechanically. Exploiting state-of-the-art three-dimensional (3D) printing, we developed a technique that not only enables the standardized clamping of soft tissues while reducing material slippage during mechanical testing, the presented solution does also increase time efficiency by reducing and simplifying sample preparation and mounting [16]. Apart from the principal improvements in sample handling and clamping, with Digital Image Correlation (DIC) a contactless strain measurement is used for data acquisition. DIC enables a precise strain measurement directly at the surface of tissues by optical co-registration of deformation further decreasing the impact of slippage on the obtained material parameters. The clamping system presented here forms an open source and accessible addition to existing environments and fixtures for biomechanical testing for soft tissue research. Specifically, this report describes an encompassing method from the design and production of equipment needed for sample preparation and clamping until mechanical testing of tissues itself.

## 2 Hardware description

Since 3D printing technology is becoming more accessible and affordable, the presented preparation and clamping system does readily facilitate biomechanical testing of soft tissues thereby collecting valuable fundamental mechanical data for future medical developments. The general idea behind the clamping system was to facilitate reproducible biomechanical data acquisition of soft tissues and to address main issues related to this approach: Material slippage, sample alignment and unequal sample dimensions were addressed. For the design of the clamping system, it was focused on modularity, printability without use of support structures, and partly on reusability. Therefore, the system has been designed to comprise of a relatively low number of parts. Identical clamping parts were designed for the opposing two sides of each clamp, thereby interlocking. A pattern of pyramids allows for form and force closure of the tissue between the two clamps when being compressed. The dimensions of the pyramids were first evaluated by investigating specimen slippage in tensile tests of human ligaments, tendons and skin [16] – the final design offers low material slippage for several types of soft tissues and a good printability in Fused Deposition Modelling (FDM). The cutting plate, preparation table and molding device form instruments for the preparation of the tissue before the mechanical testing. Holder arms and mounting adapters were designed for transferring the fragile samples from the preparation table in an aligned condition into the testing device.

• 3D printed clamps, templates and tables are designed to perform mechanical testing of soft tissues reducing material slippage and improving standardization.
• The field of use includes a broad variety of soft materials and is not limited to biological soft tissues of human origin. The clamping system can moreover be used for the standardized testing of many other soft materials.
• Key aspects of interest for scientists in medical research as well as materials engineering are the low cost, easy-to-manufacture design, the level of standardization, which comes with uniform and reproducible sample geometries and the possibility to perform high-throughput experiments with biological tissues.

## 3 Design files

The specifications are shown in Table 1 and a design files summary is given in Table 2. All electronic files are available at https://doi.org/10.17632/wbyswm62vv.3 including the computer-aided design (CAD) files and the stereolithography (STL) model files ready for printing as well as a 5-minutes instruction video explaining the complete setup. All necessary materials for printing are explained in the following section and a description for the use of each part is given in Section 5.

**Table 1:** Specifications table.

Hardware name	3D printed soft tissue clamping system
Subject area	Engineering and Material Science
Hardware type	Biological sample handling and preparation
Open Source License	CC-BY-4.0
Cost of Hardware	2.74 €
Source File Repository	https://doi.org/10.17632/wbyswm62vv.3

**Table 2:** Design files summary.

**Design file name**	**File type**	**Open source license**	**Location of the file**
Clamp wih flat pyramids	CAD & STL	CC-BY-4.0	https://doi.org/10.17632/wbyswm62vv.3
Clamp with sharp pyramids	CAD & STL	CC-BY-4.0	https://doi.org/10.17632/wbyswm62vv.3
Cutting plate bottom	CAD & STL	CC-BY-4.0	https://doi.org/10.17632/wbyswm62vv.3
Cutting plate top	CAD & STL	CC-BY-4.0	https://doi.org/10.17632/wbyswm62vv.3
Holder arm	CAD & STL	CC-BY-4.0	https://doi.org/10.17632/wbyswm62vv.3
Molding device bottom	CAD & STL	CC-BY-4.0	https://doi.org/10.17632/wbyswm62vv.3
Molding device top	CAD & STL	CC-BY-4.0	https://doi.org/10.17632/wbyswm62vv.3
Mounting adapters	CAD & STL	CC-BY-4.0	https://doi.org/10.17632/wbyswm62vv.3
Preparation table	CAD & STL	CC-BY-4.0	https://doi.org/10.17632/wbyswm62vv.3
Standardized soft tissue tensile testing	Video	CC-BY-4.0	https://doi.org/10.17632/wbyswm62vv.3

## 4 Bill of materials

All parts of the clamping system can be 3D printed and used without purchasing any additional components. Table 3 gives an overview of the quantities needed for one complete clamping system (2nd column) and about the costs for printing (4th column). For this study, polylactic acid (PLA) material from Verbatim (Premium PLA; Verbatim, Charlotte, USA) was used for all rigid parts and thermoplastic polyurethane (TPU) from Ultimaker (TPU 95A; Ultimaker B.V., Geldermalsen, The Netherlands) for all flexible parts. The material costs are derived from a retail price of the filaments of € 0.025/g for PLA and for TPU of € 0.093/g.

**Table 3:** 3D printable parts and used materials.

**Part**	**Qty**	**Cost per unit – Euro**	**Total cost – Euro**	**Manufacturer of filament**	**Material type**
Clamp with flat pyramids	4	€ 0.10	€ 0.40	Verbatim	Polymer (PLA)
Clamp with sharp pyramids	4	€ 0.09	€ 0.36	Verbatim	Polymer (PLA)
Cutting plate bottom	1	€ 0.13	€ 0.13	Verbatim	Polymer (PLA)
Cutting plate top	1	€ 0.06	€ 0.06	Verbatim	Polymer (PLA)
Holder arm	2	€ 0.60	€ 1.20	Ultimaker	Polymer (TPU)
Molding device bottom	1	€ 0.01	€ 0.01	Verbatim	Polymer (PLA)
Molding device top	1	€ 0.01	€ 0.01	Verbatim	Polymer (PLA)
Mounting adapters	4	€ 0.08	€ 0.32	Ultimaker	Polymer (TPU)
Preparation table	1	€ 0.25	€ 0.25	Verbatim	Polymer (PLA)
*Sum*			*€ 2.74*		

## 5 Build instructions

**Fig. 1:**
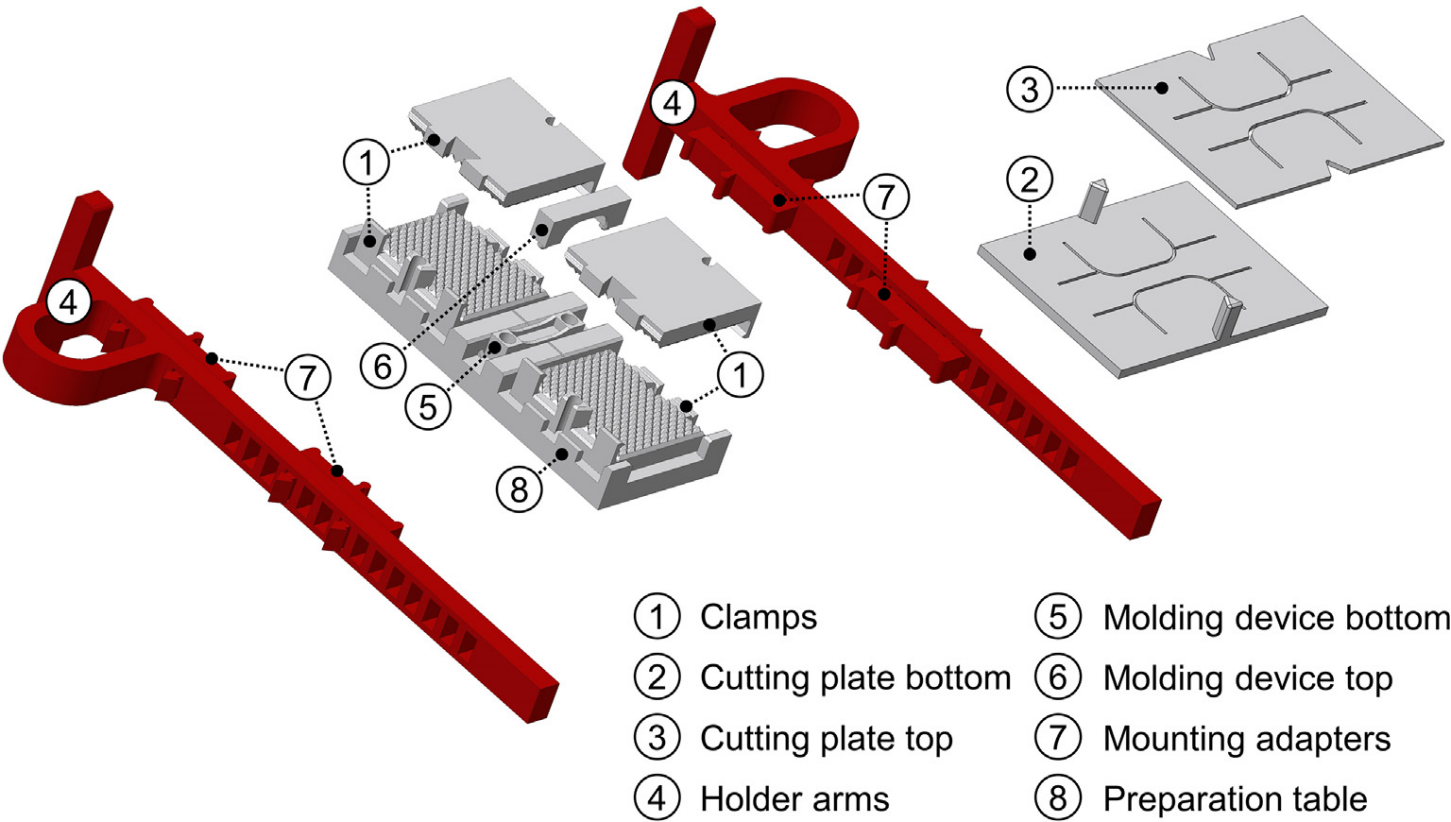
Overview – all parts from the clamping system for standardized tensile testing can be 3D printed. Parts printed in polylactic acid (PLA) are depicted grey while parts printed in thermoplastic polyurethane (TPU) are depicted in red. (For interpretation of the references to colour in this figure legend, the reader is referred to the web version of this article.)

The clamps and preparation utensils shown in Fig. 1 were designed using a CAD software (Creo 4.0; PTC software, Needham, MA, USA) with special focus on modularity and printability without the use of support structures.

• To make the resulting files accessible to a 3D printer, they first need to be exported in a tessellated file format. All files provided in Section 3 were already exported to the STL file format in high resolution and can be downloaded in this format. In order to produce highly accurate models, a customized export was used (angle control close to 0). Refining the tessellation of the models increases the file size and therefore the pre-processing time.
• The components are printed using the FDM technique. In the given case, a dual extrusion printer (Ultimaker 3 Extended; Ultimaker B.V., Geldermalsen, The Netherlands) was used. Polymer filaments are melted and deposited to the build plate layerwise. The tessellated models from the STL file format need to be sliced before printing. The recommended software for Ultimaker printer is the Ultimaker Cura slicing application.
• To print a model, it needs to be opened within the application and placed into the required position. All supplemented STL files are already oriented in the recommended orientation for printing without any support and can be printed with commercially-available filaments. For optimal results the application of a rigid material with 100% infill is strongly recommended for the clamps. The applicability of PLA for mechanical testing with the clamps has been confirmed in several studies by our group [6], [16], [17], [18]. PLA is also used for the preparation table and templates due to good printability and low warping. TPU is recommended for holder arms and mounting adapters as it is an elastic material and therefore easy to attach to and remove from the clamps. A summary of print settings is given in Table 4.

**Table 4:** Settings for 3D printing.

Printer	Any 3D printer with a heated build plate (Ultimaker 3 or S5 recommended)
Materials	PLA, TPU
Nozzle	Brass, diameter: 0.4 mm

**Settings for clamps (**flat**,sharp** sharo**)**	
Material	PLA
Layer height	0.15 mm
Infill density	100%
Wall thickness	1.00 mm
Support	no
Build plate (+adhesion)	Glass bed + glue stick + (optional: in case of adhesion problems) brim of PLA or brim of TPU*
Build plate temperature	60 °C

**Settings for all other PLA-parts (cutting plate, preparation table, molding device)**	
Material	PLA
Layer height	0.15 mm
Infill density	15%
Wall thickness	1.00 mm
Support	no
Build plate (+adhesion)	Glass bed + glue stick
Build plate temperature	60 °C

**Settings for holder arms + mounting adapters**	
Material	TPU
Layer height	0.20 mm
Infill density	15%
Wall thickness	0.76 mm
Support	no
Build plate (+adhesion)	Glass bed
Build plate temperature	No heating required

*a brim of TPU in dual extrusion slightly improves adhesion as the shifting of PLA parts at the build plate is additionally prevented and warping reduced. Moreover, it simplifies post-processing compared to a PLA brim, because TPU can be easily peeled off from PLA.
• As soon as the slicing of the model has concluded, it can be sent to the printer to initiate the printing process. Once the print has finished, (if applicable) all brim structures can be removed and the printed parts are ready to be used.

## 6 Operation instructions

A visual step-by-step instruction is also given in the supplemented video explaining the complete setup for mechanical testing.

**Fig. 2:**
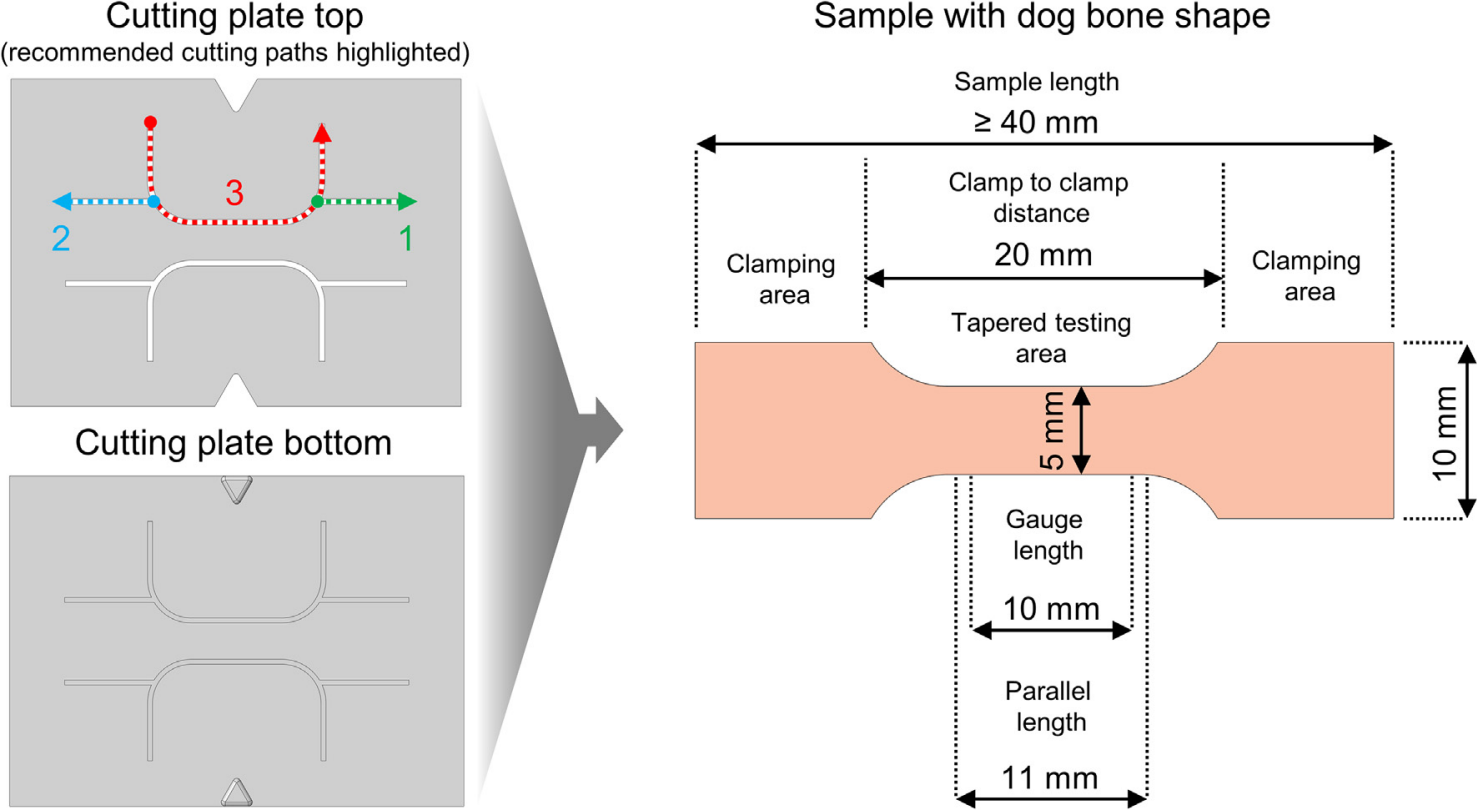
Cutting plate for dog bone-shaping of biological soft tissues as well as the final dimensions of the soft tissues after preparation. The recommended paths for cutting (from point to arrow) by a scalpel are highlighted in green (1 = first path), blue (2 = second path) and red (3 = third path) alongside dotted lines. After cutting the first side of the sample, the template is rotated and cutting is repeated for the opposite paths. (For interpretation of the references to colour in this figure legend, the reader is referred to the web version of this article.)
• To trim the samples into a standardized shape the following instruments (as presented in Fig. 3) are recommended: one pair of straight scissors, a number 3 hand piece equipped with, e.g. a size 15 scalpel, one scalpel with a pointy blade, two forceps of different sizes and the printed cutting template consisting of bottom and top part.

• To prepare the samples for the tensile tests and to create an optimal testing environment, the following step is recommended: After retrieving the samples, water content of unfixed tissues should be adapted to the tissue-dependent *in-vivo* value to increase the comparability of results by minimizing the influence of the tissues’ water content on the obtained biomechanical parameters. This can be conducted by exposing the samples to osmotic stress using a sugar-based solution and a semi-permeable membrane as described by Hammer et al. [19].
• The complete preparation and mounting process should be kept short to prevent dehydration of the sample. Additional hydration steps during the preparation process should be considered if necessary.
• The given cutting template can be used for standardized shaping of biological soft tissues into a dog bone shape (recommended, e.g. for ligaments or skin samples – note that not all biological tissues are well suited for dog bone-shaping, e.g. in case of human flexor or extensor tendons this can initiate an intrinsic sample failure, causing a non-physiological failure pattern). The dog bone shape was adapted from the ISO 527–2:2012 standard [3] with a change in the aspect ratio (2:1). The reasoning behind the low aspect ratio is the limited availability of human tissues which lowers the overall sample length in contrast to a (large enough) cross-sectional area, which can be determined with certain reliability. The cutting template as well as the resulting sample dimensions are presented in Fig. 2. Furthermore, a recommended combination of cutting paths by a scalpel is highlighted on the top plate of the cutting device.

**Fig. 3:**
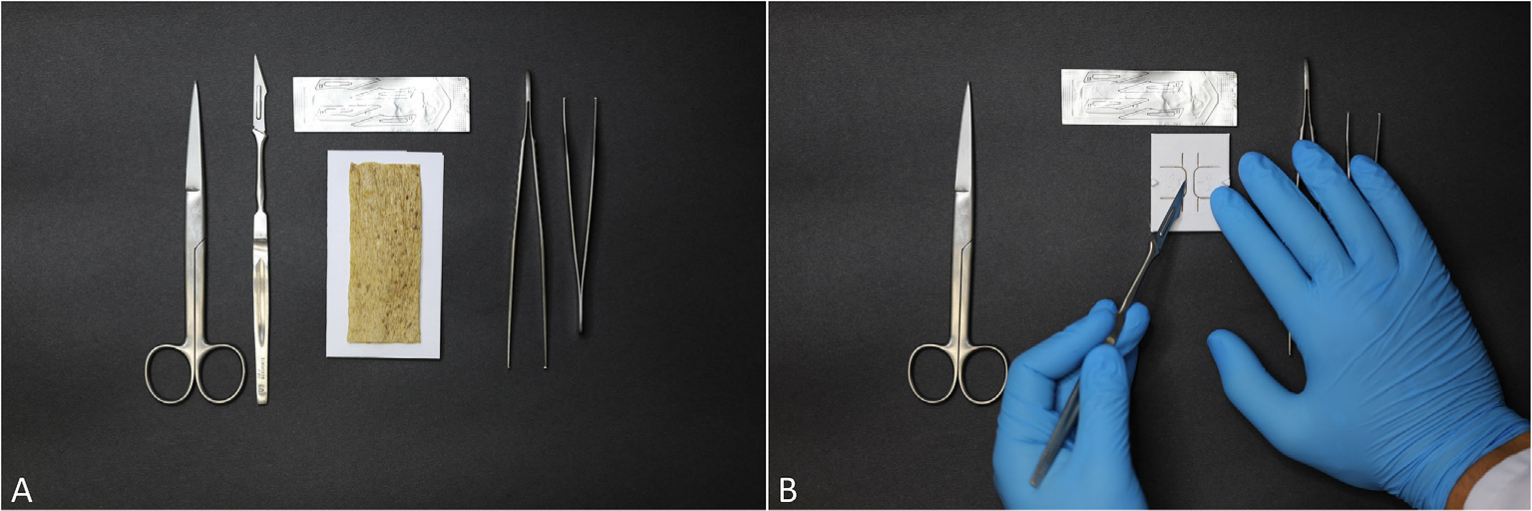
A) Instruments required for the preparation and biological soft tissue (from left to right: scissors, scalpel, blade, human/biological sample, two forceps), B) Trimming of the samples with a cutting template into a dog bone shape.
1. First, the specimen is trimmed to a size fitting inside the template using the scissors. Then it is placed on the cutting plate (Fig. 3A) and mounted with the upper part of the cutting template.
2. Using a scalpel, the sample can be trimmed into a dog bone shape adapted from the ISO 527–2 standard [3] to increase the likelihood of the sample failing in the tapered area (Fig. 3B).
• The specimen is now prepared to be clamped and the cross section can be molded for further data acquisition using the preparation table as depicted in Fig. 4.

**Fig. 4:**
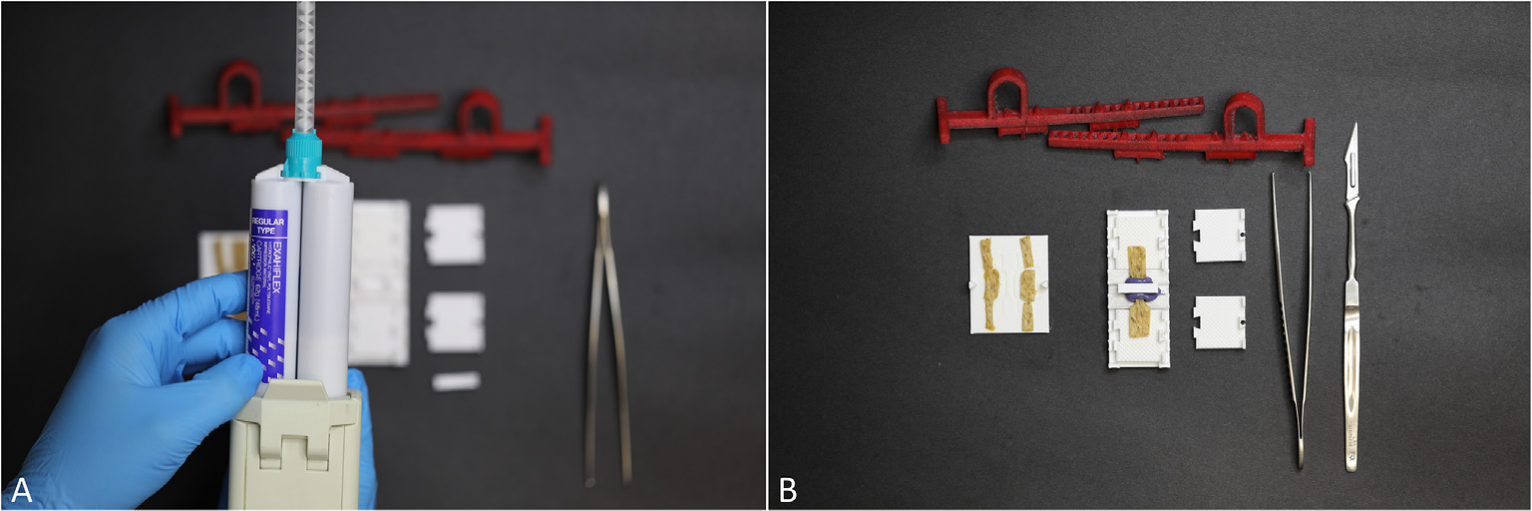
A) For determination of the specimen’s cross-sectional area a dental impression material is used. B) The trimmed sample is placed on the clamps on top of the preparation table as well as the molding device which helps forming the cast.
1. Two clamps are placed on each side of the preparation table and the bottom part of the molding device in the middle of the provided groove.
2. Following this, the sample is placed on top and fastened by two additional clamps. Clamps are fastened with the pyramids facing the sample for an optimal fixation and keep together by a clip mechanism when assembled.
3. For determining the cross-sectional area, the molding device can be used with a suitable impression material, such as dental impression material (here: polysiloxane material; medium-bodied, Exahiflex; GC Corporation, Tokyo, Japan). For this purpose, one part of the device is filled with the impression material and the other part is clipped to the bottom one.
• There are two different clamp designs which are suitable for different applications:
a) Clamps with flat pyramids: This design was found to be more suitable for tensile tests of thinner soft tissue samples.
b) Clamps with sharp pyramids: This design is well suited for thicker samples [16].
• The impression material needs to harden before carefully cutting and removing it from the sample (use of curved scissors is recommended from own experiences). The molds can now be digitally analyzed (e.g., Measure 2.1d software; DatInf, Tübingen, Germany) to precisely determine the cross-sectional area.
• The TPU holder arms are now attached to the clamped sample, which is placed on the preparation table. The arms facilitate and expedite the positioning step of the sample in the testing machine and can be adjusted to different clamp to clamp distances.
• In case of intended use of DIC for strain measurements, the application of a speckle pattern to the sample, e.g. using a small sieve and a black colored pencil, is recommended.

Mechanical testing

• After sample preparation and the attachment of the clamping system the mounted sample is placed in the testing machine as shown in Fig. 5. In this study, a universal testing machine (Zwick Roell 20 kN AllroundLine Table-Top Z020 and 2.5 kN Xforce P load cell; Ulm, Germany) was used for uniaxial tensile testing.

**Fig. 5:**
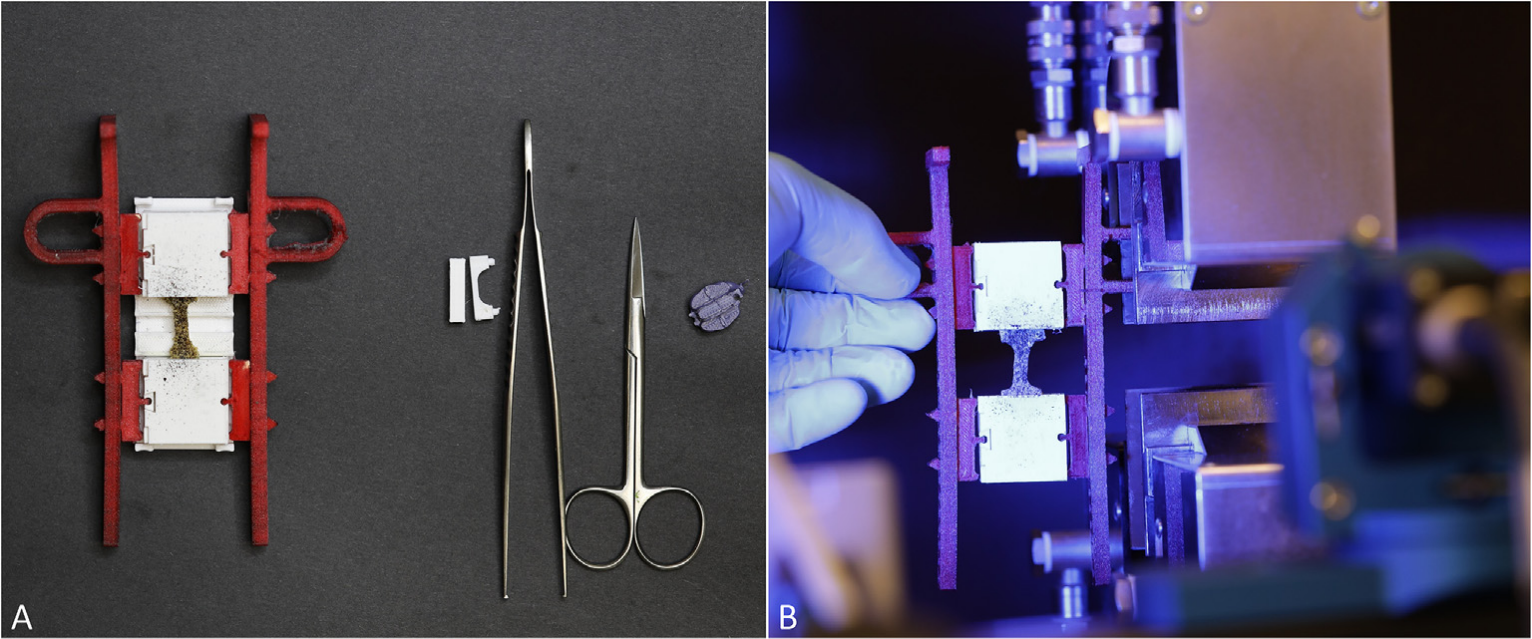
A) After mounting the flexible holder arms it is recommended to speckle the sample if using a digital image correlation-system is intended. B) The sample is placed within the testing machine by the flexible holder arms.
1. The T-profiles of the holder arms are placed on top of the gripping jaw inserts of the testing machine.
2. After fastening the gripping jaws of the testing machine, the flexible holder arms of the clamping system must be removed by pulling the lugs before starting the test.
3. It is recommended to conduct a strain-controlled tensile test in order to obtain comparable stress–strain curves [20].
• A testing machine with pneumatic gripping jaws is recommended to provide a constant gripping force during testing and increase user-friendliness. The maximum tensile force applied should not exceed 1 kN when metal inserts are used as the interface between 3D printed PLA clamps and the gripping jaws of the testing machine to prevent failure of the polymer material during the test. Using rubber-coated inserts or inserts with a central mold does increase the maximum applicable load significantly.

(Optional) DIC can be used to obtain more accurate deformation data as it enables measuring the strain directly at the sample’s surface excluding deformation of the testing machine’s components, mechanical play and material slippage [21].

**Fig. 6:**
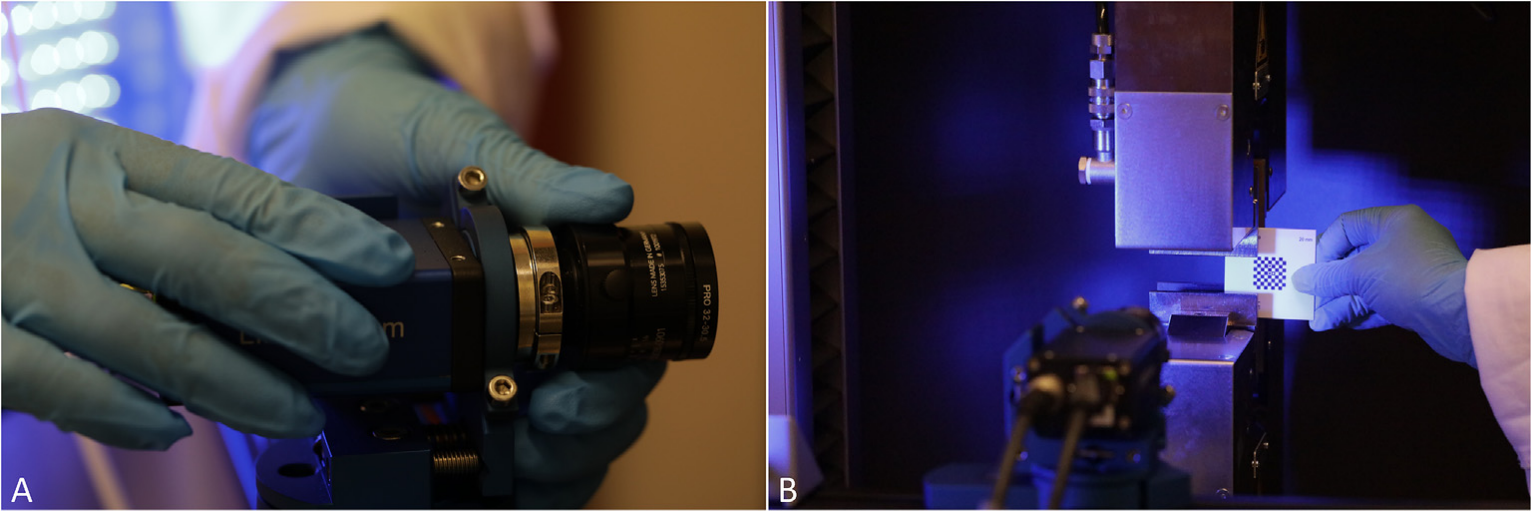
Digital image correlation-setup using a Limess Q400 system by Dantec Dynamics. A) First, the camera is placed allowing the recording of the specimen’s area of interest. After focusing the camera, B) the digital image correlation-system is calibrated using a standardized calibration target.
• After placing the camera perpendicular to the specimen axis, focusing and calibrations are carried out to obtain images at a level of quality, which can be used for image-based analyses. In this given setup, a GI-WMB-9x9-calibration target is used, allowing a maximum global residuum of 0.025 mm. It is important to solidly mount the camera on a tripod or to the testing machine to avoid movements, which then require re-calibration.
• The simultaneous recording of force data by the testing machine and image data by the DIC system requires a synchronization of both systems with the same time base. In this study, the testing machine was connected to the DIC system by an input/output card (NI PCle-6321; National Instruments, Austin, USA). Digital outputs were used for trigger events (start and end of image recording). The force-readings of the testing machine (as well as the displacement of the crosshead, which was only used as a control function) were transferred via an analogue voltage signal with a suitable resolution to the DIC system. Recording of the DIC data was automatically started by trigger events as soon as the pre-load was reached and stopped when the break of the sample occurred.
• The obtained full-field image data can be further processed in the DIC software to calculate local strain distributions at the sample surface as well as under inclusion of the force readings of the testing machine a stress-strain response of each sample. This data can be used to adapt the parameters of constitutive models, to validate computational models and for experimental studies to investigate the dependency of the mechanical behavior, e.g. on age or sex. For quantitative analyses, virtual extensometers can be defined in the area of sample parallel length to calculate an engineering strain while the sample is stretched. Furthermore, under the inclusion of the cross-sectional area, an engineering stress can be calculated from the force readings. The synchronized data can be used to calculate engineering stress–strain curves, which are useable for the evaluation of mechanical properties. In case of a simplified linear elastic material model elastic modulus, Poisson’s ratio, ultimate tensile strength or a strain at failure can be evaluated.

• DIC analysis can be carried out with any commercially-available system. Here, a Limess DIC system (Q400; Limess, Krefeld, Germany and Istra4D; Dantec Dynamics, Skovlunde, Denmark) has been used as shown in Fig. 6. One or two cameras generate 2D or 3D data, respectively [22]. In the given setup, one camera was oriented perpendicular to the sample surface for soft tissue tensile testing to record time-dependent full-field image data during mechanical testing.

## 7 Validation and characterization

The new 3D printed clamping design has meanwhile been applied for several studies, predominantly involving large-scale experiments on human tissues for the purpose of mapping the human body biomechanically.

• These tissues involved: scalp, mucosa, dura mater, skeletal muscle and muscle fascia, ligaments and tendons in both static and dynamic testing scenarios [6], [16], [17], [18].
• A first study aimed at quantifying electron microscopically the effects of the clamps on tissue integrity and could show that failure predominantly occurred in the areas of parallel measurement length and not in the clamping regions. Furthermore, this study demonstrated that a specimen throughput of several samples per hour (depending on the tested tissue) can easily be reached in a highly standardized manner even if the testing involved image correlation, and that the clamping design provides sufficient grip to allow even for fatigue tests [16]. This finding opens the opportunity for large-scale experiments with human/biological tissues without exerting them to the risk of degradation in a high-volume high-throughput manner.
• Clamps with flat pyramids were found out to offer suitable interlock with thinner samples like tendons or ligaments, with an initial thickness (before compression by clamps) up to approximately 2 mm. For thicker samples like human skin or scalp, clamps with sharp pyramids offer more suitable interlocking up to an initial thickness of about 5 mm. Nevertheless, preliminary tests show that the complete clamping system is scalable up to 200% before printing without any problems, which allows larger samples and sample thicknesses, necessary for example when testing certain animal tissues.
• Mechanical tests using the 3D printed clamps could furthermore show that despite the large standard deviations observed in ligaments and tendons such as the iliotibial band, measurement variation can be decreased vastly by minimizing inaccuracies resulting from the clamping procedure itself or material slippage in combination with tissue heterogeneity, allowing to assess for sex and age differences of the biological soft tissues [23].
• Further experiments on the same tissue type could provide evidence that tissue mechanics are largely governed by their intrinsic water content, in line with their micromechanical behavior [24]. Previous studies demonstrated sex-, age- and site-related differences in the load-deformation properties of various soft tissues [17].
• Highly slippery material such as Thiel-embalmed biological tissues could be tested using tissue clamps with sharp pyramids - a study assessing the effects of anatomical fixation on soft tissues of the head could for the first time show that certain embalming techniques with presumed 'lifelike' haptic and optical properties have deviating mechanical properties, yielding them unsuitable for biomechanical validation studies if accurate properties resembling the vital state are required [25].
• The combined experience from these studies have shown that material testing of up to four specimens per hour can be reached even when combined with DIC - depending on the type of soft tissue tested as well as the number of researchers conducting the tests. Further to this, settle differences in the mechanical properties could be observed despite the well-documented natural variation of human soft tissues, proving this method to be suitable for hypothesis-based research questions and studies correlating tissue mechanics on a multi-scale level.
• Several other studies also confirm the applicability of 3D printed clamps for the mechanical testing of biological soft tissues:
  o In a recent study of Wood et al. 3D printed clamps were used for tendon testing at low strains to obtain Young’s modulus and Poisson’s ratio. Similar values compared to experiments with cryo-jaws confirm the applicability of using 3D printed clamps [26].
  o A similar method by Grgić et al. substantiates the benefits of using 3D printing for biomechanical testing based on our method presented here. The printed clamps were found to be suitable for biomechanical testing of tendons, as the mechanical behavior of the area of interest was not affected by the clamping. Furthermore, the accessibility of 3D printed parts, the low costs and the ease of use were found to be advantageous compared to other methods [27].
  o In a recent study, Jiang et al. compare a novel 3D printed clamping system with conventional tissue clamping methods. A combination of 3D printed serrated clamps with needle fixtures was found to be appropriate for tensile testing of abdominal wall tissue [28].

## 8 Limitations

• The complete clamping system presented here has been shown to be reusable several times depending on the requirements for aseptic or sterile conditions of the experiments – only the clamps are limited to an average reuse of about four times before mechanical failure of the clip mechanisms [16]. PLA was initially chosen as a printing material for the clamps due to its printability and biodegradability. A limitation is formed by the relatively low melting point of PLA which prevents the repeated application of thermal autoclave sterilization techniques [16]. Therefore, it is recommended reusing printed parts from PLA and TPU (clamps, cutting plate and preparation table, mounting adapters) only within one given study, especially when handling potentially infectious fresh tissues and to dispose them immediately afterwards. Nevertheless, the application of engineering plastics with a higher strength and a higher melting point could possibly resolve those limitations in the future. Furthermore, alternative sterilization techniques, e.g. with irradiation or chemical sterilization might be possible to clean the parts for reuse in an aseptic environment.
• Dog bone-shaping is recommended for the standardized testing of, e.g. ligaments or skin. Not all biological soft tissues are well suited for dog bone-shaping.

## Declaration of Competing Interest

The authors declare that they have no known competing financial interests or personal relationships that could have appeared to influence the work reported in this paper.

## References

[1] DIN EN ISO 6892-1: 2017-02, Metallische Werkstoffe_- Zugversuch_- Teil_1: Prüfverfahren bei Raumtemperatur (ISO_6892-1:2016); Deutsche Fassung EN_ISO_6892-1:2016, Beuth Verlag GmbH, Berlin, 2017.

[2] DIN EN 658-1: 1999-01, Hochleistungskeramik_- Mechanische Eigenschaften von keramischen Verbundwerkstoffen bei Raumtemperatur_- Teil_1: Bestimmung der Eigenschaften unter Zug; Deutsche Fassung EN_658-1:1998, Beuth Verlag GmbH, Berlin, 1999.

[3] DIN EN ISO 527-2: 2012-06, Kunststoffe_- Bestimmung der Zugeigenschaften_- Teil_2: Prüfbedingungen für Form- und Extrusionsmassen (ISO_527-2:2012); Deutsche Fassung EN_ISO_527-2:2012, Beuth Verlag GmbH, Berlin, 2012.

[4] ASTM D3039/D3039M–17 Test Method for Tensile Properties of Polymer Matrix Composite Materials 2017 ASTM International West Conshohocken, PA

[5] International Organization for Standardization, Rubber, vulcanized or thermoplastic – Determination of tensile stress-strain properties, Beuth Verlag GmbH, Berlin 83.060, 2017.

[6] Zwirner J., Ondruschka B., Scholze M., Schulze-Tanzil G., Hammer N. (2019). Mechanical and morphological description of human acellular dura mater as a scaffold for surgical reconstruction. J. Mech. Behav. Biomed. Mater..

[7] Griffin M., Premakumar Y., Seifalian A., Butler P.E., Szarko M. (2016). Biomechanical characterization of human soft tissues using indentation and tensile testing. J. Vis. Exp..

[8] Humphrey J.D. (2003). Review Paper: Continuum biomechanics of soft biological tissues. Proc. R. Soc. Lond. A.

[9] Fung Y.-C. (1993).

[10] Bowser J.E., Elder S.H., Rashmir-Raven A.M., Swiderski C.E. (2011). A cryogenic clamping technique that facilitates ultimate tensile strength determinations in tendons and ligaments. Vet. Comp. Orthop. Traumatol..

[11] Ng B.H., Chou S.M., Krishna V. (2005). The influence of gripping techniques on the tensile properties of tendons. Proc. Inst. Mech. Eng. H.

[12] Sichting F., Steinke H., Wagner M.-F.-X., Fritsch S., Hädrich C., Hammer N. (2015). Quantification of material slippage in the iliotibial tract when applying the partial plastination clamping technique. J. Mech. Behav. Biomed. Mater..

[13] Shi D., Wang D., Wang C., Liu A. (2012). A novel, inexpensive and easy to use tendon clamp for in vitro biomechanical testing. Med. Eng. Phys..

[14] Wu J.Z., Brumfield A., Miller G.R., Metheny R., Cutlip R.G. (2004). Comparison of mechanical properties of rat tibialis anterior tendon evaluated using two different approaches. Bio-Medi. Mater. Eng..

[15] Saunders M.M. (2015).

[16] Scholze M., Singh A., Lozano P.F., Ondruschka B., Ramezani M., Werner M., Hammer N. (2018). Utilization of 3D printing technology to facilitate and standardize soft tissue testing. Sci. Rep..

[17] Falland-Cheung L., Scholze M., Lozano P.F., Ondruschka B., Tong D.C., Brunton P.A., Waddell J.N., Hammer N. (2018). Mechanical properties of the human scalp in tension. J. Mech. Behav. Biomed. Mater..

[18] Zwirner J., Scholze M., Waddell J.N., Ondruschka B., Hammer N. (2019). Mechanical properties of human dura mater in tension – An analysis at an age range of 2 to 94 Years. Sci. Rep..

[19] Hammer N., Huster D., Fritsch S., Hädrich C., Koch H., Schmidt P., Sichting F., Wagner M.-F.-X., Boldt A. (2014). Do cells contribute to tendon and ligament biomechanics?. PLoS One.

[20] Joseph R. (2010).

[21] Pan B., Qian K., Xie H., Asundi A. (2009). Two-dimensional digital image correlation for in-plane displacement and strain measurement: A review. Meas. Sci. Technol..

[22] Schreier H., Orteu J.-J., Sutton M.A. (2009).

[23] Zwirner J., Babian C., Ondruschka B., Schleifenbaum S., Scholze M., Waddell N.J., Hammer N. (2019). Tensile properties of the human iliotibial tract depend on height and weight. Med. Eng. Phys..

[24] Lozano P.F., Scholze M., Babian C., Scheidt H., Vielmuth F., Waschke J., Ondruschka B., Hammer N. (2019). Water-content related alterations in macro and micro scale tendon biomechanics. Sci. Rep..

[25] Zwirner J., Scholze M., Ondruschka B., Hammer N. (2019). Tissue biomechanics of the human head are altered by Thiel embalming, restricting its use for biomechanical validation. Clin. Anat..

[26] Vella Wood M., Casha A., Gatt A., Formosa C., Chockalingam N., Grima J.N., Gatt R. (2019). 3D printed clamps to study the mechanical properties of tendons at low strains. Phys. Status Solidi B.

[27] Grgić I., Ivandić Ž., Šotola D., Kozak D., Karakašić M. (2018). Thread inspired 3D printed clamps for in vitro biomechanical testing. Proc. TEAM.

[28] Jiang M., Lawson Z.T., Erel V., Pervere S., Nan T., Robbins A.B., Feed A.D., Moreno M.R. (2020). Clamping soft biologic tissues for uniaxial tensile testing: A brief survey of current methods and development of a novel clamping mechanism. J. Mech. Behav. Biomed. Mater..

